# Mitigating dengue transmission in Africa: the need for *Wolbachia*-infected mosquitoes' rollout

**DOI:** 10.3389/fpubh.2024.1506072

**Published:** 2024-12-16

**Authors:** Samson T. Ogunlade, Adeshina I. Adekunle, Emma S. McBryde

**Affiliations:** ^1^Australian Institute of Tropical Health and Medicine, James Cook University, Townsville, QLD, Australia; ^2^Defence Science and Technology Group, Department of Defence, Melbourne, VIC, Australia; ^3^Centre for Clinical Research, Faculty of Medicine, The University of Queensland, Brisbane, QLD, Australia

**Keywords:** dengue, *Wolbachia*, mosquitoes, rollout, Africa

## Introduction

Dengue fever is a mosquito-borne viral disease that poses a significant public health concern globally (1–3). The disease is primarily transmitted by *Aedes aegypti* mosquitoes and the range of clinical manifestations vary from flu-like symptoms (4, 5) to more serious conditions such as dengue haemorrhagic fever and dengue shock syndrome (6). The dengue virus (DENV) infects about 400 million people yearly, of which 50–100 million of those become symptomatic, with over 20,000 deaths (3).

Dengue notifications are increasing in Africa (1, 2). The continent's tropical and subtropical climatic conditions create a conducive breeding environment for mosquitoes and hence, contribute to the spread of the virus (1, 4, 7, 8). While recent statistics show that there were 15.7 million reported dengue infections in 2010, recent studies (1, 7, 8) show that dengue cases are on the rise in Africa. This situation presents an increasing threat to public health systems already under pressure from other infectious illnesses.

Traditionally established vector control methods such as the use of insecticide, emptying or covering water-filled containers and eliminating mosquito breeding sites have had limited success in curbing the spread of dengue (9, 10). This calls for experimental and innovative strategies to combat the disease effectively (9). One promising approach—the *Wolbachia*-based approach, involves the deployment of *Wolbachia*-infected mosquitoes into the wild mosquito population. This technique has shown great potential in reducing dengue transmission (11, 12). While the *Wolbachia*-based technique has demonstrated highly positive results in mitigating DENV, it is not always successful—*Wolbachia* strategies may struggle in high temperature settings, because some mosquitoes infected with *Wolbachia* (such as *w*Mel strain) are unable to transmit *Wolbachia* maternally to their offspring and establish themselves under high temperatures (13, 14). Therefore, using thermally tolerant strains may be beneficial in establishing *Wolbachia* infections in mosquitoes especially in regions with high heat conditions (14–16). Although *Wolbachia*-infected mosquitoes have been rolled out in different countries such as Brazil (17), Colombia (18) in South America; Indonesia (19), Taiwan (20), Viet Nam (21), Thailand (22), Malaysia (23), India (24) in South Asia; Northern Queensland in Australia (16, 25); and the United States of America (26), there is arguably no deployment yet made in Africa.

In this opinion piece, we discuss the urgent need to begin the implementation the *Wolbachia*-based strategy in Africa as a complimentary control strategy to other existing control methods on the continent. This may be useful in contributing to the mitigation or eradication of DENV especially in high endemic settings.

## The burden of dengue in Africa

Several factors are contributing to the rise of Dengue fever in Africa (1, 2, 8). With a tropical and subtropical climate, regions of Africa have ideal breeding grounds, but DENV is also known to increase with urbanization (1, 8). Many countries in Africa have had rapid urbanization and population growth in the setting of inadequate public health infrastructure (2, 27, 28). The continent's tropical and subtropical climates create ideal breeding conditions for *Aedes* mosquitoes, increasing the spread of the virus (29). Furthermore, rapid urbanization, population growth, and inadequate public health infrastructure contribute to the increasing dengue burden (30). According to the World Health Organization, dengue is now endemic in many African countries, posing a significant challenge to public health systems already strained by other infectious diseases (31).

## Established and experimental vector control measures

Established measures of vector control include chemical and environmental control methods such as insecticide use, emptying or covering water-filled containers and clearing of mosquitoes' breeding sites (9). Some of these methods of controlling dengue vectors have limitations (32). While insecticide use is widely practiced and initially effective with estimates suggesting up to a 50% reduction in mosquito population (10), it may lead to increased resistance in *Aedes* mosquito populations, thereby reducing its long-term efficacy (33). Although, chemical control methods such as larvicides and pyrethroids are estimated to reduce adult population by around 80% (34), the effects are not sustained and mosquito populations generally revert to baseline levels within few days to weeks (35). In addition, despite their effectiveness, chemical control methods could cause harmful environmental and health problems such as pollution and contamination of aquatic animals and irritation to humans (9). Efforts to eliminate mosquito breeding sites through community participation and environmental management such as regular covering or emptying of water-filled containers are crucial but often faced with challenges in implementation and sustainability (36). These limitations highlight the need for novel and more sustainable interventions to control dengue transmission, particularly in Africa where constraints are presented by limited resources, heterogeneous environmental conditions.

## *Wolbachia*-based strategy: a promising solution

*Wolbachia* is an endosymbiotic bacterium found in many insect species (9, 37). When introduced into *Aedes aegypti* mosquitoes, *Wolbachia* reduces the mosquitoes' ability to transmit dengue viruses (38). *Wolbachia*-infected mosquitoes have lower life-span (39) and reduced viral replication (9), effectively decreasing the likelihood of virus transmission to humans (38). Field trials in several countries, such as Australia, Brazil, and Indonesia, have demonstrated significant reductions in dengue incidence following the release of *Wolbachia*-infected mosquitoes (17, 19, 25). As a demonstration, studies in Northern Queensland, Australia, revealed that dengue incidence has decreased by roughly 96% (CI: 84–99%) (25). In the Brazilian city of Niteroi, dengue incidence has decreased by an estimated 69% (CI: 54–79%) (17). Similarly, the release of mosquitoes carrying the *Wolbachia* bacteria averted the number of dengue cases in Indonesia by ~86.2% [Uncertainty Interval (UI): 32.2 – 99.9%] (13). Another recent study conducted in Medellin, Colombia evaluated the prevalence and distribution of *Wolbachia*-carrying *Aedes* mosquitoes 2 years after rollout completion. The authors found that *Wolbachia* prevalence remained lower than expected, with only 33.5% of mosquito pools testing positive 2 years after rollout, indicating significant geographical variance (40). Similar study carried out in Rio de Janeiro, Brazil (41) also showed that *w*Mel-*Wolbachia* reduced dengue incidence by just 38%. Recently, studies have shown that *Wolbachia*-based methods could be temperature-sensitive as *Wolbachia* infections in mosquito may not be established via maternal transmission in high temperature regions (13, 15, 42). This information suggests that sustaining stable *Wolbachia* prevalence may be difficult, most likely due to environmental conditions, emphasizing the necessity for continuing monitoring and future reintroduction (15, 16). Overall, these findings provide strong support for the implementation of *Wolbachia*-based strategy in other areas, particularly Africa, by highlighting its efficacy in a variety of ecological and epidemiological contexts, notwithstanding some concerns about sustaining the incidence of *Wolbachia* in these settings.

## The case for *Wolbachia* in Africa

The successful introduction of *Wolbachia*-infected mosquito programs can change the paradigm for dengue control in Africa. This strategy allows for the following benefits:

- Sustainable: Once introduced, *Wolbachia*-infected mosquito populations can thrive and replace themselves without additional releases forever eliminating dengue transmission (43).
- Eco-friendly: *Wolbachia*-based technique is environmentally safe and does not have any impact on non-target species compared to chemical insecticides (44).
- *Wolbachia* presence: *Wolbachia*-based programs can incorporate and benefit local communities, promoting acceptance and compliance (11).
- Cost effectiveness: While initial costs for *Wolbachia*-based programs may be high and expensive, after several years these burdens can recede, and dengue-related healthcare costs would decline, hence it is a cost-saving intervention (45).

## Challenges and recommendations

Despite the potential success of *Wolbachia*-based strategies, the future of *Wolbachia*-infected mosquitoes' releases in Africa remains challenging (46). Challenges include logistics, the regulations and community engagement and orientation (16, 38). To overcome these challenges, the following recommendations are proposed:

- Collaboration: Governing bodies, international organizations, and research institutions must collaborate to facilitate the deployment of *Wolbachia* programs to combat viral diseases. The success of these programs will depend critically on the sharing of resources such as demographic data and information.
- Regulatory support: African countries must create a robust regulatory framework for the successful deployment and surveillance of *Wolbachia*-infected mosquitoes (47). This involves making sure safety inspections are conducted and ensuring the people is convinced (47).
- Community engagement: Effective communication strategies and support must be developed to educate communities about the benefits of *Wolbachia* and to address any misconceptions or fears (48, 49). Community involvement is essential for the acceptance and success of the program (48).
- Variation in temperature: Although *Wolbachia* has shown great success in mitigating DENV, high temperate conditions could dampen its effectiveness in establishing *Wolbachia* and becoming self-sustaining (13, 15, 42, 50). Therefore, *Wolbachia* rollout in Africa would require continuous thermally resistant Wolbachia strain to boost effectiveness and efficacy in reducing arboviruses such as dengue especially in high temperate African regions (1, 14).
- Funding and Resources: *Wolbachia*-based programs need ongoing funding and resources for their initial deployment and consistent continuous supervision before they may eventually become self-sustaining (11, 51). It will be essential to have both governmental and private sector investment for successful deployment of *Wolbachia*-infected mosquitoes (1, 19, 45).

## Discussion

The introduction of *Wolbachia*-infected mosquitoes into African countries is a novel strategy that has the potential to significantly reduce the dengue epidemic (1, 46). However, putting such a program into action necessitates giving careful thought to many different factors.

Firstly, a careful assessment of the ecological effects of releasing mosquitoes infected with *Wolbachia* is necessary (52). Despite *Wolbachia*'s natural occurrence and relative safety, it is important to monitor its entry into new settings to avoid unintentionally harming nearby ecosystems (9, 11). Extensive research is required to guarantee that the advantages outweigh any possible hazards (38). It is critical to investigate the ecological dangers and uncertainties of introducing *Wolbachia*-infected mosquitos (9, 44) as variations in dengue virus serotypes across Africa may potentially have an impact on *Wolbachia*'s efficacy (53).

Secondly, *Wolbachia* program success is largely reliant on community acceptance and engagement (36, 48, 49). African cultures can differ greatly in their attitudes about modern technologies and mosquitoes (54). Customized communication plans that consider regional traditions and community issues are crucial (48). Building trust and gaining support for the program can be facilitated by involving community leaders and stakeholders in the planning and implementation process (11, 36).

Thirdly, integrating *Wolbachia* programs with existing vector control measures can enhance their effectiveness (9, 55). A more effective defense against dengue transmission can be achieved by combining *Wolbachia*-based program with established vector control techniques like environmental management and insecticide application (55). Coordination between various public health initiatives such as the African Centers for Disease Control and Prevention and the World Health Organization Africa Region will be necessary to optimize resources and achieve the best feasible *Wolbachia*-infected mosquitoes rollout outcomes (56).

Fourthly, studies have shown that *Wolbachia* infections in mosquitoes are sensitive to high temperature conditions (13, 15, 16). These conditions can reduce the effectiveness in establishing Wolbachia-carrying mosquitoes, resulting in an increase in dengue transmission (42, 50). Considering Africa's diverse climate, variations in temperature may have an impact on *Wolbachia*'s performance, indicating the need for specialized strategies such as employing *Wolbachia* strains that are more heat-resistant (42, 50, 57).

Finally, sustainable resources and funding are essential for the long-term success of *Wolbachia* programs (58). Initial investments are required for research, development, and implementation of *Wolbachia*-based techniques (15, 58). In addition, continuous support and monitoring is needed for the maintenance of the mosquito population (47). International funding, public-private and non-governmental organization partnerships can play a vital role in ensuring that these programs are adequately resourced (32, 45, 51). *Wolbachia*-based mosquito control is a long-term solution and while it is more expensive than insecticides and the removal of mosquito breeding sites, it reduces dengue transmission without the need for repeated interventions after establishment (9, 32). Success in Brazil and Indonesia has demonstrated that community engagement through collaborations with local leaders, educational orientation programs, and open communication is critical to establishing public confidence and support for *Wolbachia* initiatives (17, 59). Adopting comparable engagement tactics in African contexts could capitalize on community networks and local expertise, increasing program acceptance and adaptability to tackle *Aedes*-borne diseases effectively and sustainably (1).

## Conclusion

Dengue fever remains a significant public health challenge in Africa, calling for creative methods of containing its spread. A safe and sustainable biological method of mitigating dengue transmission in Africa is via mosquitoes carrying the intra-cellular endosymbiont *Wolbachia* bacteria. It is imperative that this strategy urgently be developed so that it can safely and sustainably be implemented in Africa. *Wolbachia* programs have the potential to become an essential component of dengue control initiatives, enhancing public health results throughout the continent by tackling barriers related to logistics, regulations, and community engagement. Africa may take the lead in implementing this ground-breaking technique to suppress the effects of dengue viral disease through corroborative efforts, regulatory support, community participation, and ongoing funding and resources.
